# Retraction Note: Numerical assessment of the influence of helical baffle on the hydrothermal aspects of nanofluid turbulent forced convection inside a heat exchanger

**DOI:** 10.1038/s41598-023-28023-2

**Published:** 2023-01-18

**Authors:** Li Yang, Shaghayegh Baghaei, Wanich Suksatan, Pouya Barnoon, Sandhir sharma, Alla Davidyants, A. S. El-Shafay

**Affiliations:** 1 School of Equipment Engineering, Shenyang Ligong University, Shenyang, 110159 Liao Ning China; 2 Department of Mechanical Engineering, Khomeinishahr Branch, Islamic Azad University, Isfahan, Iran; 3 Faculty of Nursing, HRH Princess Chulabhorn College of Medical Science, Chulabhorn Royal Academy, Bangkok, Thailand; 4 Department Chitkara Business School, Faculty-Business Management, University- Chitkara University, Punjab, India; 5 Department of Propaedeutics of Dental Diseases, Sechenov First Moscow State Medical University, Moscow, Russia; 6 Department of Mechanical Engineering, College of Engineering, Prince Sattam Bin Abdulaziz University, Alkharj, 16273 Saudi Arabia; 7 Mechanical Power Engineering Department, Faculty of Engineering, Mansoura University, Mansoura, 35516 Egypt

Retraction of: *Scientific Reports* 10.1038/s41598-022-06049-2, published online 10 February 2022

Editors have retracted this Article.

After publication, concerns were raised about authorship and description of author contributions. The Editors requested the authors to provide raw original data and explanations regarding the contributions, but found the response provided by the Authors insufficient. The Authors were also not able to provide the data in the format that would allow for the confirmation of its veracity (i.e. including sufficiently detailed meta-data). Additionally, it appears that references 57-63 appear to be unrelated to the research described in this article. The Editors therefore no longer have confidence in the reliability of the data presented in this Article. Shaghayegh Baghaei disagrees with the retraction. The Editors were not able to obtain current contact details for the other Authors.

